# Characterization of the complete chloroplast genome of *Pterocarya macroptera* var. *delavayi* (Juglandaceae)

**DOI:** 10.1080/23802359.2021.1910085

**Published:** 2021-04-05

**Authors:** Hua Yan, Jisi Zhang, Jianfei Ye, Chunce Guo, Xiaoguo Xiang

**Affiliations:** a Forestry College, Jiangxi Provincial Key Laboratory for Bamboo Germplasm Resources and Utilization, Jiangxi Agricultural University, Nanchang, PR China; b College of Chemistry and Life Sciences, Anshan Normal University, Anshan, PR China; c State Key Laboratory of Systematic and Evolutionary Botany, Institute of Botany, Chinese Academy of Sciences, Beijing, PR China; d Jiangxi Province Key Laboratory of Watershed Ecosystem Change and Biodiversity, Institute of Life Science and School of Life Sciences, Nanchang University, Nanchang, PR China

**Keywords:** *Pterocarya macroptera* var. *delavayi*, chloroplast genome, phylogenetic analysis

## Abstract

In this study, the complete chloroplast genome sequence of *Pterocarya macroptera* var. *delavayi* was reported and characterized. The chloroplast genome is 160,168 bp in length, and consists the typical quadripartite structure, a pair of inverted repeats (IRs, 26,007 bp) separated by a large single-copy region (89,701 bp) and a small single-copy region (18,453 bp). A total of 136 unique genes were predicted, including 88 protein-coding genes, 40 tRNA genes, and 8 rRNA genes. The GC content of the chloroplast genome is 36.2%. Phylogenetic analysis confirmed the close relationship between *Pterocarya* and *Juglans*.

*Pterocarya macroptera* var. *delavayi* distributes in western Hubei, western Sichuan, and northwest Yunnan of China (Lu et al. 1999). It is a riparian dominant tree, and its bark can be used as fiber raw material (Lu et al. 1999; Ying and Chen 2011). *P. macroptera* var. *delavayi* is a variety of *P. macroptera*, which is morphologically different from *P. macroptera* var. *macroptera* by its mature leaves with exclusively solitary trichomes (Song et al. 2020). Based on RAD-seq data, Song et al. (2020) showed that the genus *Pterocarya* is monophyletic and close to *Juglans*. *P. macroptera* var. *delavayi* was sister to the other two varieties (*P. macroptera* var. *macroptera* and *P. macroptera* var. *insignis*) (Song et al. 2020).

Here, the complete chloroplast genome of *P. macroptera* var. *delavayi* was determined, annotated, and analyzed. The sample was collected from the Botanical Garden Edinburgh (UK) (55°56′54.56″N, 3°1′57.59″ W) by Jianfei Ye. The DNA sample (RBGE 2019-08-27) was deposited in the Institute of Life Science, Nanchang University (JXU). Total genomic DNA was extracted from silica gel-dried leaves using the modified CTAB method (Doyle 1987) and sequenced using MGI MGISEQ-2000 High-throughput Sequencing Set. In total, 3 Gb of 150-bp paired-end raw reads were generated and used for chloroplast genome assembly. Trimmomatic version 0.39 (Bolger et al. 2014) was used to organize and trim overrepresented sequences for getting the clean reads. The clean reads were assembled by using GetOrganelle version 1.5 (Jin et al. 2020). The chloroplast genome of *P. macroptera* var. *delavayi* was annotated used Geneious version 9.05 (http://www.geneious.com/) with *P. stenoptera* (NC_046428) as reference. The annotated complete chloroplast genome of *P. macroptera* var. *delavayi* was deposited in GenBank (the accession number MW194257).

The complete chloroplast genome of *P. macroptera* var. *delavayi* is 160,168 bp in length, with a large single-copy region (LSC) of 89,701 bp, a small single-copy region (SSC) of 18,453 bp, and a pair of inverted repeat regions (IRs) of 26,007 bp, and forms a typical quadripartite structure. A total of 136 genes were annotated from the chloroplast genome of *P. macroptera* var. *delavayi*, including 88 protein-coding genes, 40 transfer RNA (tRNA) genes, and 8 ribosomal RNA (rRNA) genes. The GC content of the chloroplast genome is 36.2%, meanwhile, the corresponding value of LSC, SSC, and IR regions is 33.8%, 29.8%, and 42.6%, respectively.

In order to analyze the phylogenetic position of *P. macroptera* var. *delavayi*, another 22 complete chloroplast genomes were retrieved from NCBI, and *Morella rubra* from Myricaceae was set as the outgroup (Figure 1). All sequences were aligned by MAFFT version 7.409 (Katoh and Toh 2010). The maximum likelihood tree and Bayesian tree were constructed using RAxML version 8.2.12 (Stamatakis 2014) and MrBayes version 3.2.6 (Ronquist and Huelsenbeck 2003), respectively. The phylogenetic tree showed that *Pterocarya* is formed a clade with the *Juglans* with high support (ML-PP = 100%, BI-PP = 1.0), and *P. macroptera* var. *delavayi* is sister to *P. tonkinensis* with strongly supported values (ML-PP = 100%, BI-PP = 1.0, Figure 1). The phylogenetic relationships among Juglandaceae were consistent with the results of Xiang et al. (2014) and Mu et al. (2020) based on chloroplast genome data.

**Figure 1.**
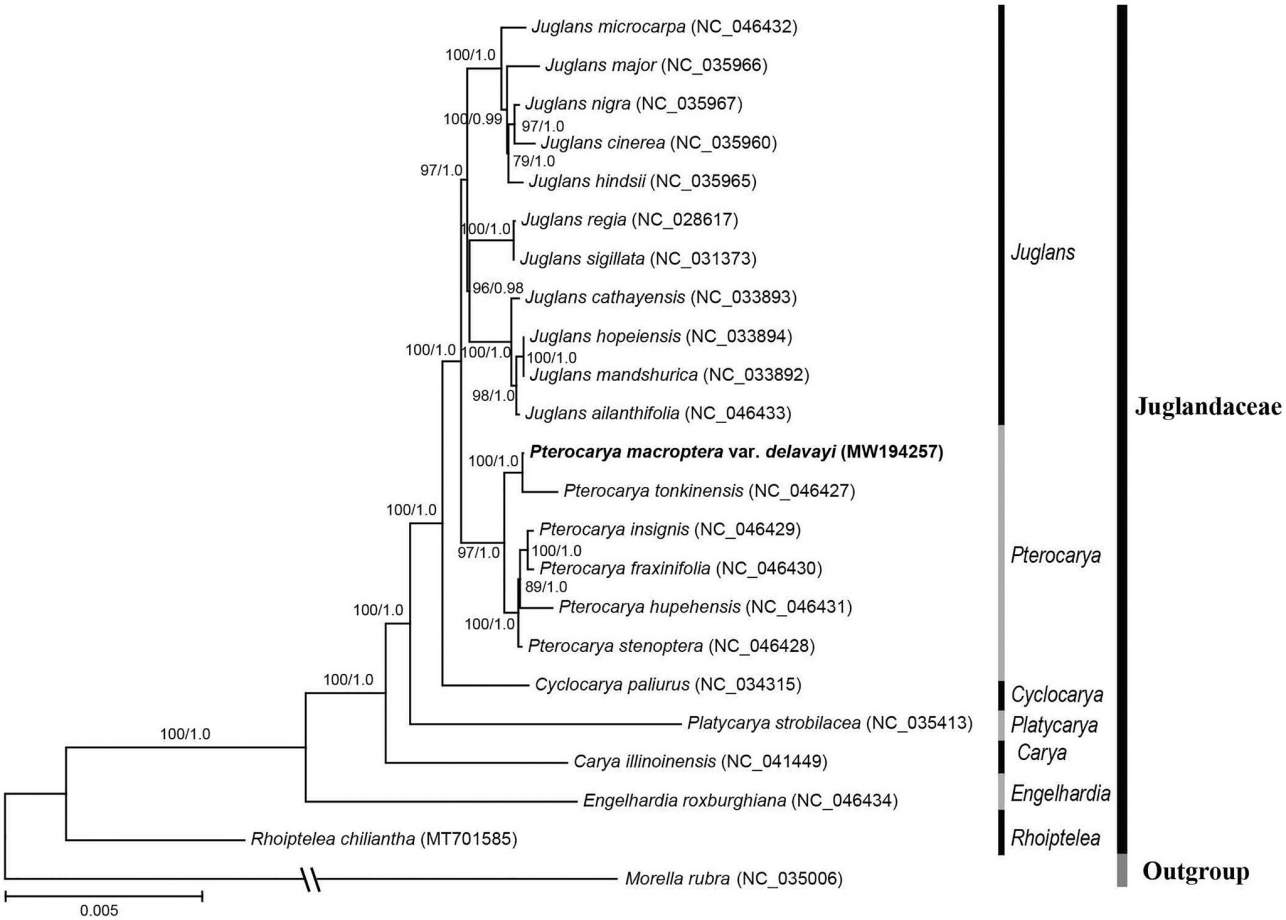
The maximum likelihood (ML) tree of 23 species inferred from the complete chloroplast genome sequences. Numbers associated with branches are ML bootstrap values and Bayesian posterior probabilities.

## Data Availability

The genome sequence data that support the findings of this study are openly available in GenBank of NCBI at https://www.ncbi.nlm.nih.gov/ under the accession no.MW194257. The associated BioProject, SRA, and Bio-Sample numbers are PRJNA692030, SRR13434420, and SAMN17304756, respectively.

## References

[CIT0001] Bolger AM, Lohse M, Usadel B. 2014. Trimmomatic: a flexible trimmer for Illumina sequence data. Bioinformatics. 30(15):2114–2120. 24695404 10.1093/bioinformatics/btu170 PMC4103590

[CIT0002] Doyle JJ. 1987. A rapid DNA isolation procedure for small amounts of fresh leaf tissue. Phytochem Bull. 19:11–15.

[CIT0003] Jin JJ, Yu WB, Yang JB, Song Y, dePamphilis CW, Yi TS, Li DZ. 2020. GetOrganelle: a fast and versatile toolkit for accurate de novo assembly of organelle genomes. Genome Biol. 21(1):241. 32912315 10.1186/s13059-020-02154-5 PMC7488116

[CIT0004] Katoh K, Toh H. 2010. Parallelization of the MAFFT multiple sequence alignment program. Bioinformatics. 26(15):1899–1900. 20427515 10.1093/bioinformatics/btq224 PMC2905546

[CIT0005] Lu AM, Stone DE, Grauke LJ. 1999. Juglandaceae. In: Wu ZY, Raven PH, editors. Flora of China, Vol. 4. Beijing, China: Science Press & Missouri Botanical Garden Press; p. 277–285.

[CIT0006] Mu XY, Tong L, Sun M, Zhu YX, Wen J, Lin QW, Liu B. 2020. Phylogeny and divergence time estimation of the walnut family (Juglandaceae) based on nuclear RAD-Seq and chloroplast genome data. Mol Phylogenet Evol. 147:106802. 32217170 10.1016/j.ympev.2020.106802

[CIT0007] Ronquist F, Huelsenbeck JP. 2003. MrBayes 3: Bayesian phylogenetic inference under mixed models. Bioinformatics. 19(12):1572–1574. 12912839 10.1093/bioinformatics/btg180

[CIT0008] Song YG, Li Y, Meng HH, Fragnière Y, Ge BJ, Sakio H, Yousefzadeh H, Bétrisey S, Kozlowski G. 2020. Phylogeny, taxonomy, and biogeography of *Pterocarya* (Juglandaceae). Plants. 9(11):1524. 10.3390/plants9111524 PMC7696814 33182441

[CIT0009] Stamatakis A. 2014. RAxML version 8: a tool for phylogenetic analysis and post-analysis of large phylogenies. Bioinformatics. 30(9):1312–1313. 24451623 10.1093/bioinformatics/btu033 PMC3998144

[CIT0010] Xiang XG, Wang W, Li RQ, Lin L, Liu Y, Zhou ZK, Li ZY, Chen ZD. 2014. Large-scale phylogenetic analyses reveal fagalean diversification promoted by the interplay of diaspores and environments in the Paleogene. Perspect Plant Ecol. 16(3):101–110.

[CIT0011] Ying TS, Chen ML. 2011. Plant geography of China. Shanghai, China: Shanghai Scientific & Technical Publishers; p. 141.

